# Palliative (farewell) culture in shared housing arrangements: The perspective of everyday nursing practice

**DOI:** 10.1007/s00391-024-02313-4

**Published:** 2024-06-04

**Authors:** Carola Walter, Katharina Lex

**Affiliations:** Institute of Nursing Science and Practice, Paracelsus Medical University, Strubergasse 21, 5020 Salzburg, Austria

**Keywords:** Nursing, Shared housing arrangement, Innovative living arrangement, Palliative care, Culture, Pflege, Ambulant betreute Wohngemeinschaft, Innovative Wohnform, Palliativversorgung, Kultur

## Abstract

**Background:**

Shared housing arrangements (SHA) are alternatives to long-term care facilities for care-dependent people. The collective perspective of nursing professionals working in SHA in dealing with death and dying is missing in recent studies. This study aimed to investigate the perspective of professionals concerning a palliative (farewell) culture in SHA.

**Methods:**

In this study two group discussions were conducted with nurses and nursing assistants working in SHA. Data were analyzed using the documentary method, with the aim of working out the professional orientation framework concerning a collective palliative culture.

**Results:**

Nurses enable a palliative (farewell) culture. This leads to the fact that hospice services are not used in these SHA. The distance to relatives as well as a short dying process or incomplete dying support can make a successful palliative culture difficult. Depending on the conscious assumption of responsibility for a palliative culture in the nursing concept of SHA, death and dying are discussed at an early stage with the relatives and care-dependent people.

**Discussion:**

The constantly progressing palliative culture in SHA is based on nurses’ experiences, general practitioners (GP) and relatives. The family carers’ role is ambiguous. If they do what they are supposed to do from the professional nurses’ point of view and are closely connected to the nurses, they are viewed positively and as enablers of a palliative culture. If family carers’ responsibilities are not communicated and they are not in close contact with professional nurses, they are viewed as opponents of a palliative culture. The GPs are seen as enablers of a palliative culture in both discussions. A timely discussion on what might happen in the end of life phase, formalized or not, helps all involved groups to be prepared.

**Supplementary Information:**

The online version of this article (10.1007/s00391-024-02313-4) contains supplementary material, which is available to authorized users.

## Abstract (de)

**Hintergrund:**

Ambulant betreute Wohngemeinschaften (abWG) sind für pflegebedürftige Personen eine Alternative zum Seniorenheim. Die kollektive Perspektive der dort tätigen Pflegenden bezüglich Tod und Sterben fehlt in aktuellen Studien. Ziel der vorliegenden Arbeit war es, die Perspektive der professionell Pflegenden hinsichtlich einer palliativen (Abschieds‑)Kultur in den Wohngemeinschaften zu untersuchen.

**Methodik:**

Es wurden zwei Gruppendiskussionen mit in abWG tätigen Pflegenden und Pflegehelfenden geführt. Die Auswertung erfolgte anhand der dokumentarischen Methode mit dem Ziel, den professionellen Orientierungsrahmen der Pflegenden in Bezug auf eine kollektive palliative Kultur herauszuarbeiten.

**Ergebnisse:**

Pflegende ermöglichen eine palliative (Abschieds‑)Kultur. Dies zeigt sich unter anderem darin, dass diese Wohngemeinschaften keine Hospizdienste in Anspruch nehmen. Distanz zu Angehörigen, ein unvorhergesehener Sterbeverlauf oder eine unvollständige Sterbebegleitung können eine gelungene palliative Kultur erschweren. Abhängig von der bewussten Übernahme von Verantwortung für eine palliative Haltung im pflegerischen Konzept der Wohngemeinschaft, werden Tod und Sterben frühzeitig mit den Angehörigen und den zu Pflegenden thematisiert.

**Diskussion:**

Pflegende in abWG leben eine sich stetig weiterentwickelnde palliative (Abschieds‑)Kultur. Basis dafür sind die Erfahrungen der Pflegenden wie auch die Rolle der Angehörigen und Hausärzte. Angehörige spielen bei der Gestaltung der palliativen (Abschieds‑)Kultur eine ambivalente Rolle. Ist die Beziehung zu den Pflegenden vor Ort eng und übernehmen sie die von der Pflege zugedachte Rolle, so werden sie im positiven Sinne als „Mitspieler“ hinsichtlich einer palliativen Kultur wahrgenommen. Bei nicht klar kommunizierter Verantwortungsübernahme durch Angehörige und bestehender Distanz zu den Pflegenden werden Angehörige als „Gegenspieler“ erlebt. Die Hausärzte wurden in beiden Gruppendiskussionen als Förderer einer palliativen Kultur wahrgenommen. Ein frühzeitiges Besprechen der letzten Lebensphase, egal ob mit oder ohne formale Verfügungen, kann alle involvierten Akteure dabei unterstützen, die bestehende palliative Kultur weiter auszubauen.

## Background

At the end of 2021 there were around 4.6 million people in need of care in Germany, almost 81% of whom were cared for at home [18]. Home care is maintained for a long time. Haumann showed that this setting is preferred when care is needed [6] and 20% of those surveyed could imagine moving into a shared housing arrangement (SHA) if their care needs increased. Only 5% would consider moving into a long-term care facility (LTCF). The reasons for moving into an LTCF or another form of assisted living include increasing age, progressive dementia and increasing care needs. In addition to these personal factors that influence the decision to move, there is a growing burden on relatives and the challenge for them to provide rising levels of care. The onset of neuropsychiatric symptoms, a deterioration in general health, and polymedication are additional factors [1, 17, 23].

The LTCFs are the most common places of relocation in Germany when care dependency arises. Alternative forms of housing still do not appear in the national statistics, although these have been promoted in Germany for years [3, 15]. Klie et al. estimated that around 3100 SHA exist in Germany [12]. The fundamental principles of SHA are autonomy and participation in an everyday living environment, including security and normality for the people living there [4, 5, 11, 12]. The target group of alternative living places, such as SHA, are care-dependent people and those with cognitive decline or dementia [2, 26]. From a professional point of view, it is evident when a person moves into a SHA that this move is usually the last and the final phase of life begins, even if this cannot be precisely determined in terms of time [14, 19].

Kojer and Heimerl [13] showed that the oldest and most care-dependent people are worthy of palliative care and should benefit from palliative care as well. A palliative culture within organizations has been mainly considered in nursing homes and development processes have been initiated [7, 22]. Studies in SHA consider death and dying exploratively or descriptively from the perspective of different actors [21, 25]. The people living in SHA, the interaction with all actors, and the simultaneous “guest status” of the nursing service raise questions about how death and dying are dealt with and the attitude towards death and dying in this form of living [12]. A palliative culture integrated into the daily life of care-dependent people seems necessary as a basis for life in SHA. How professional nurses practice this culture is the subject of this article. It is about a practiced palliative (farewell) culture.

This qualitative study aimed to identify, analyze, and subsequently reconstruct everyday professional knowledge and professional nurses’ collective patterns of action as a group. The guiding research question was: “to what extent do SHA enable a palliative (farewell) culture in everyday nursing practice?”

## Methods

The research-based interest lies in different forms of everyday knowledge and the conditions for the actors. Everyday knowledge is embedded in specific contexts, is therefore indexical and can only be understood in a situation-dependant context. Applying the documentary method according to Bohnsack enables exploration of the relevant systems of the groups involved [20]. The practice of nurses in SHA is reconstructed [9]. Care services that started providing care in SHA by 2014 at the latest were included. They were also asked to have experience in dealing with death and dying to be able to be involved in the discussion. The relevant care services were taken from a list of SHA in Bavaria from 2017. A total of 11 outpatient Bavarian care services were contacted in December 2017 with a personal letter, including the information letter and the informed consent for the study. After 1–2 weeks, a telephone inquiry was made, and further verbal explanations were given in response to queries from the care services. Convenience sampling was used. In total, six nurses and nurse assistants from two care services in three SHA were recruited for the group discussions at the beginning of 2018. The group discussions took place on site and were recorded using a recording device. Written consent was obtained for participation. The guided interviews were conducted by CW. The guiding was based on the current literature, and consultation with an initiator of SHA regarding its relevance for nurses was undertaken [5, 12, 21]. In addition, two pilot interviews with experienced nurses from nursing homes and palliative care units supplemented the preparation to ensure the necessary structure while maintaining the greatest possible openness and flexibility [20]. After each group discussion, CW prepared a postscript to record the situational context [16]. The individual group discussions were transcribed in MAXQDA version 12 (Verbi Software GmbH, Berlin, Germany), which the researcher (CW) anonymized [20]. Data analysis was based on the documentary method [9]. At the beginning of the analysis, the thematic course of the discussion was worked out. A formulating interpretation for the presentation of the thematic sequence and the structuring of the content aspects followed. In the third step, data analysis was directed from “what was said” to “how it was said”. The formal structure was reconstructed, making different orientations of participating nurses visible and enabling homologies to be sought both within and outside the case [9]. The researcher conducting the study (CW) has many years of experience as a nursing professional and a nurse educator. Information on the researcher was given when requested in the information and clarification letter. One person (CW) analyzed the data and discussed them with others during the colloquia in the master’s program and with the supervisor. The consolidated criteria for reporting qualitative research (COREQ) formed the basis for preparing the article [24].

## Results

The central orientations reconstructed from the group discussions are presented to initiate a conversation about palliative (farewell) culture.

## Characteristics of care services/nurses

Both participating care services have been working in SHA since they were established. In one care service, the nurses work only in the SHA; in the other, they also work in the outpatient sector outside the SHA. Due to illness, only two people took part in the group discussion in Care Service 1 and four people were involved in the group discussion in Care Service 2. All participating persons’ professional experience working in SHA was between 9 months and 7 years. Table 1 shows the participating nurses with their qualifications. Group discussion 1 lasted 40 min and 17 s; the second group discussion lasted 51 min and 13 s.

**Table 1:** Care services and participating persons

Care Services	Care Service 1	Care Service 2
Number of SHA	1 SHA	2 SHA
Qualification of participating persons	1 Geriatric nurse/nursing management 1 Social nurse assistant	1 Geriatric nurse 2 Registered nurses 1 Nurse assistant

*SHA* shared housing arrangements

## Orientations

### “… but that’s different palliative care with us because” (GD2: ZN 39 ff)

**Fig. 1:**
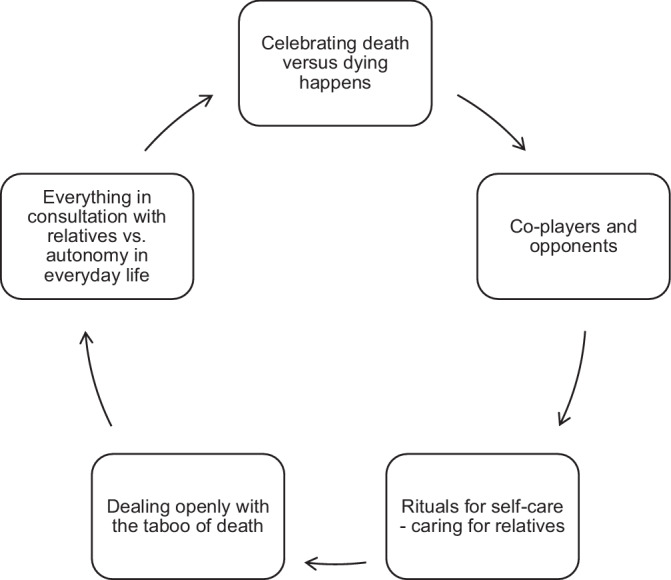
Different orientations of a palliative (farewell) culture in SHA

This opening statement from one of the participants (Fw) in group discussion 2 (GD2) demonstrates right from the start of the discussion that the forms of palliative care differ in SHA from their nursing perspective. These differences are reflected in the orientation framework developed in the analysis in Fig. 1.

### Celebrating death versus dying happens

Aw: “Up to now, we’ve been celebrating all these years (2) yes, someone was dying, but we didn’t celebrate it like we are now with her” (GD1: ZN 194-195). The core aspect of “celebrating death” was evident in both group discussions. Nursing professionals need to be able to support people in SHA during the dying process and to do this with or without relatives. This support is reflected in performing rituals. Detailed narrative elaborations of positively experienced dying processes can also be found in other passages of the group discussions. On the other hand, unpredictable processes cannot be planned and, therefore challenge professionals.

### Co-players and opponents

**Fig. 2:**
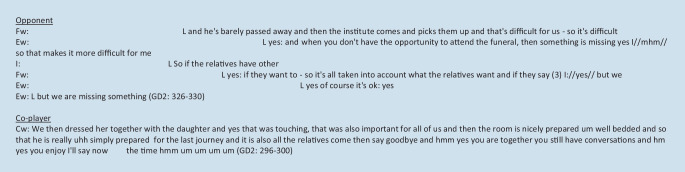
Quotations—relatives as co-players or opponents

Co-players and opponents include all those working in SHA and their relatives. Co-players are people who constructively influence a palliative (farewell) culture. Opponents include groups of people who set themselves apart, making a palliative (farewell) culture more difficult. Both groups of nurses formulate challenges in dealing with relatives and the resulting orientation dilemma for themselves. The distance is reflected in the exclusion of professionals from the dying process and the subsequent support. Although nurses are aware that everything that relatives want must be observed professionally, the exclusion is difficult to bear as the high density of interaction in this passage shows. This is where different normative concepts of “good dying” and coping with grief clash. The orientation of relatives as opponents is contrasted with the counter-horizon of relatives as co-players. This shows the potential of good end of life care in the joint organization of the dying process and the care of the deceased person after death. Figure 2 gives examples of relatives as co-players or opponents.

**Fig. 3:**
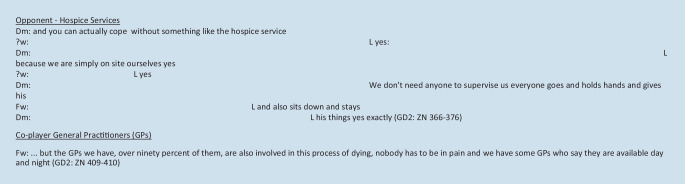
Quotations—hospice services as opponents and general practitioners as co-players

Both groups exclude palliative care and hospice services from the SHA, with the relatives being involved in positive dying processes. Towards the end of group discussion 1, the exclusion of these was supplemented by the comment, “Maybe the relatives trust us so much, that’s why no palliative care team is needed (2) this idea just came to me” (GD1: ZN 475-476). The relatives’ trust in the care provided in the SHA as a starting point for exclusion can be seen as an essential factor in caring for the dying. However, the possibility of spatial and personal closeness in contrast to a nursing home is also emphasized. At the same time, the feeling of being monitored by the hospice service is mentioned. Support from this service is not discussed. One nurse confirmed the existing possibility of good support in the SHA. This interactive dialogue reveals the possibility of being present and providing independent palliative care. The hospice service and palliative care team are seen as implicit opponents and excluded. In contrast, the general practitioners are seen as co-players. Figure 3 gives examples of the discussions.

Both explained orientation frameworks are influenced by the remaining three orientations: rituals for self-care and caring for relatives, dealing openly with the taboo of death, and everything in consultation with relatives versus autonomy in everyday life. This article used the first two orientations to explain the palliative (farewell) culture from the everyday perspective of nurses.

## Discussion

Nurses in SHA are generally open to a palliative (farewell) culture, practice it, and constantly develop it further. This is based on the fundamental principles of SHA “autonomy”, “participation”, and “normality”, which aim to enable life until the end [11]. From the nurses’ perspective, relatives and GPs are relevant for implementing a palliative (farewell) culture. Relatives can act as co-players and opponents, depending on whether the collaboration is successful. Collaboration relies on the presence of relatives, the relationship between relatives and nurses, and their different perspectives. This is also shown by Reitinger et al., who see communication and relationship building with relatives as a challenge [21]. Relatives, therefore, feel left alone with the responsibility [21]. If this feeling is shared with passively acting nurses, this can challenge cooperation even more. An insufficiently clarified assumption of responsibility may exacerbate the distance to the relatives described in the study. The results of the existing studies, therefore, coincide here, and the findings of this study can deepen the organization of relationships and communication based on the orientations presented. A longer dying phase makes it easier for nurses and relatives to exchange information and prepare for it together. Similar results concerning good cooperation with other stakeholders were found in another study [25]. The collaboration with GPs is perceived as positive. From the nurses’ point of view, the care options in SHA provide the necessary proximity to provide palliative care as the only care service in normal dying processes. The hospice service or the palliative care team are therefore not included, although they are well known [25]. In contrast, Worch et al. recommended increased cooperation with hospice services in their longitudinal study [27]. The hospital as an opponent was not explicitly presented, as this perception of nurses was only clearly expressed in one group; however, the nurses’ statements reflected the results of the other two studies, and there appears to be barely improved cooperation [21, 25].

Volunteering, an essential form of support in SHA, was not mentioned in either of the two group discussions [12]. The need for early communication becomes apparent in the cooperation between the various players in the palliative (farewell) culture. Living wills as an option for self-determination are viewed ambivalently. While in one SHA, it is mandatory to present a living will when moving in, the other care service states that these are rarely available but even if they are available their validity is doubted and communication with relatives is used instead. This corresponds to the “dialogue-based decision-making” (p. 896) that patients want, as they do not see the living will as a definitive document [10]. Despite this positive attitude of nurses toward the palliative (farewell) culture, it is visible that a structured approach in the sense of advance care planning has not (yet) started [8].

### Limitations

Limitations of the study result from the regulations of a master’s thesis. Many steps in the research process were carried out by the researcher alone; however, these were discussed as part of the supervision and the research colloquia. The inclusion of only two groups also limits the results; however, the participants had a wide range of experience in SHA and other fields of nursing care so that this experience could be incorporated into the discussions. Due to the time constraints for the qualification work, further discussions were impossible. Reasons for nonparticipation in the study by care services were the time of year, inquiry before Christmas and the high nursing staff workload. The documentary method pursues a possible type of formation via case-comparative abstraction, which serves to generalize research results [10]. This could not be done due to the small number of group discussions.

## Conclusion for practice

- Challenges arise in interacting with relatives and the involvement of hospice services/palliative care teams. Creating structures and processes that enable successful interaction appears to be expedient.
- Experience with death and dying grows with years of working in SHA. By actively incorporating concepts such as advanced care planning, nurses and care services can initiate processes to enable a sustainable palliative (farewell) culture and develop it further.

### Supplementary Information

Orientierungen und Interviewausschnitte auf Deutsch

## Declarations

For this article no studies with human participants or animals were performed by any of the authors. All studies mentioned were in accordance with the ethical standards indicated in each case. Ethical approval: a certificate stated no ethical concerns. It was issued on 7 December 2017 by the Ludwig-Maximilians-Universität München, project number 17-707.
