# Supplementary material for: Palliative (farewell) culture in shared housing arrangements: The perspective of everyday nursing practice
Source: Z Gerontol Geriatr. 2024 Jun 4;57(4):284–9. doi: 10.1007/s00391-024-02313-4 (PMC11208262; doi:10.1007/s00391-024-02313-4)
Supplement: Supplementary file 1 — Orientierungen und Interviewausschnitte auf Deutsch [file 391_2024_2313_MOESM1_ESM.docx]

Orientierungen und Interviewausschnitte auf Deutsch

**
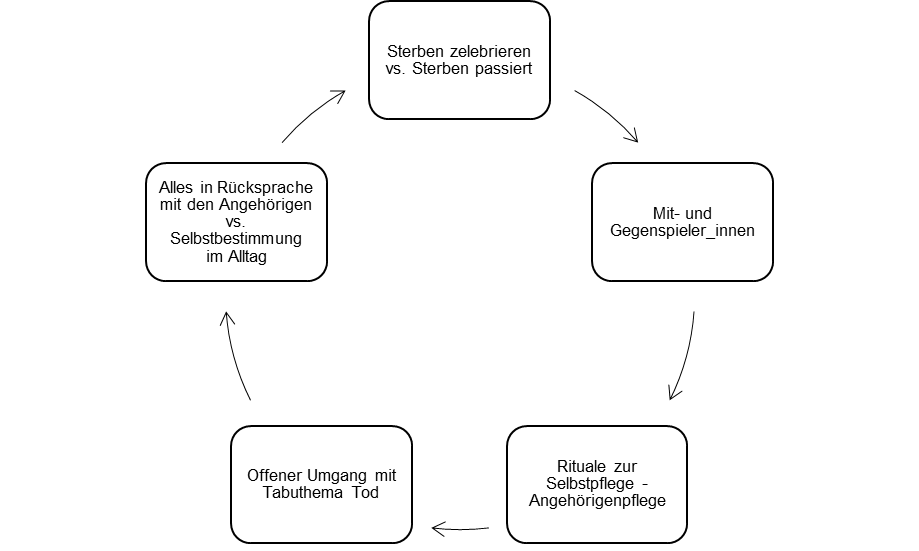
**

Abbildung 2: Orientierungen „Palliative (Abschieds-)Kultur“ in abWG (eigene Darstellung)

**Sterben zelebrieren versus Sterben passiert**

Aw:„bisher ham wir die ganzen Jahre(2) ja da war halt zwar jemand im Sterben, aber wir ham da nicht so zelebriert wie jetzt bei Ihr“ (GD1: ZN 194-195)

**Mit- und Gegenspieler:innen**

Fw: L und er ist kaum verstorben dann ist schon das Institut ab und holt sie ab und das ist etwas was uns schw- also es schwer

Ew: L ja: und wo man dann auch nicht die Möglichkeit hatte zur Beerdigung zu gehen zur Bestattung da fehlt dann schon was ja I//mhm// also das machts mir schwieriger

I: L also wenn die Angehörigen andere

Fw: L ja: wenn die an- also es wird alle beachtet was die Angehörigen wollen und wenn die sagen (3) I://ja// aber wir

Ew: L ja ist natürlich ok: ja

Ew: L aber uns ja fehlt dann was (GD2: 323-228)

Cw: wir haben dann gemeinsam mit der Tochter angezogen und ja das war rührend auch das war das war für uns alle wichtig und dann wird das Zimmer schön hergerichtet ähm gut gebettet und so dass er wirklich ähh für die letzte Reise einfach dann fertig gemacht wird und es ist auch alle Angehörigen kommen dann verabschieden sich und hmm ja man ist miteinander man führt Gespräche noch und hm ja man genießt sag ich jetzt mal noch die Zeit hmm ähm ähm (GD2: 294-301)

„*Vielleicht vertrauen uns die Angehörigen so, deswegen kein Palliativteam (2) kam mir grad mal so*“ (GD1: ZN 475-476)

Dm: und man kann eigentlich auf sowas wie den Hospizdienst auch verzichten

?w: L ja:

Dm: L weil wir sind selber einfach vor Ort ja

?w: L ja

Dm: wir brauchen niemanden der nur mitschaut jeder geht einmal hin und hält die Hand und gibt noch sein

Fw: L und setzt sich auch hin und bleibt

Dm: L seine Sachen dazu ja genau (GD2: ZN 366-376)

Fw: … aber die Ärzte die wir haben so über neunzig Prozent tragen auch diesen Prozess des Sterbens mit keiner muss Schmerzen haben und wir haben manche Hausärzte die sagen wir sind Tag und Nacht da (GD2: ZN 409-410)
